# Strategic use of β-mannanase and xylanase β-glucanase preparation in maize-based diets to improve broiler performance

**DOI:** 10.1016/j.aninu.2025.09.003

**Published:** 2025-11-01

**Authors:** Eunjoo Kim, Mingan Choct, Anna Fickler, Guilherme A.M. Pasquali, Leon Hall, Tamsyn M. Crowley, Nishchal K. Sharma

**Affiliations:** a School of Environmental and Rural Science, University of New England, Armidale, NSW 2350, Australia; b BASF SE, 67056 Ludwigshafen, Germany; c Poultry Hub Australia, University of New England, Armidale, NSW 2350, Australia

**Keywords:** Broiler, β-Glucanase, β-Mannanase, Growth performance, Non-starch polysaccharides, Xylanase

## Abstract

Poultry feed contains significant amounts of undigested components, driving the search for innovative strategies to improve their utilization. This study aimed to evaluate the effects of β-mannanase, alone or in combination with a xylanase and β-glucanase preparation (XG), on growth performance, nutrient and energy utilization, and the gastrointestinal environment of broilers fed maize-based diets. A total of 384 mixed-sex Cobb 500 d-old broilers were assigned to 4 treatments for 35 days in a 2 × 2 factorial arrangement, with β-mannanase (with or without) and XG (with or without) as factors. Each treatment included eight replicate pens of 12 birds each, totalling 96 birds per treatment. During d 0 to 10, dietary supplementation of β-mannanase increased weight gain (*P* = 0.009) and improved feed conversion ratio (FCR) (*P* < 0.001) compared to the unsupplemented control. During d 11 to 21, β-mannanase improved FCR (*P* = 0.039), and β-mannanase and XG interacted for weight gain (*P* = 0.008), where β-mannanase or XG alone increased weight gain compared to the unsupplemented control but there were no further improvements achieved via their simultaneous use. During d 22 to 35, XG supplementation increased weight gain (*P* = 0.003) and improved FCR (*P* = 0.032). During d 0 to 35, XG improved FCR (*P* = 0.019), and XG and β-mannanase tended to interact for FCR (*P* = 0.071), with either of the enzymes improving FCR compared to the unsupplemented control but with no further improvements when added in tandem. During d 0 to 35, XG and β-mannanase interacted for weight gain (*P* = 0.026), where XG improved weight gain in the absence of β-mannanase but there were no further improvements achieved via their simultaneous use. Dietary β-mannanase increased total tract retentions of dry matter (*P* = 0.001), insoluble non-starch polysaccharides (NSP, *P* = 0.010), total NSP (*P* = 0.010), calcium (*P* = 0.020), apparent metabolizable energy (AME, *P* < 0.001) and nitrogen-corrected AME (AME_n_, *P* < 0.001). Dietary β-mannanase and XG interacted for acetic acid (*P* = 0.034) and total short-chain fatty acid (SCFA, *P* = 0.044) concentrations in the caecal contents, where β-mannanase increased their concentrations in the absence of XG, but there were no further improvements when both enzymes were used in tandem. In conclusion, the supplementation of β-mannanase in a maize-based diet improved growth performance in the early stages of broiler development by enhancing the utilization of NSP, nutrients, and energy. On the other hand, XG improved growth performance in the grower-finisher stages; however, in this study, the combination of β-mannanase and XG did not produce synergistic effects on growth performance or nutrient and energy utilization in broilers fed maize-based diets.

## 1 Introduction

Poultry feed contains a significant proportion of undigested components, which can negatively impact production efficiency, profitability, and sustainability. Broilers consuming a maize-based diet excrete approximately 31.5% of nutrients at 35 d of age, with one-third being fibre, which includes non-starch polysaccharides (NSP) plus lignin, followed by proteins and others (Kim et al., 2022). This suggests an opportunity to develop nutritional strategies to maximize the utilization of the undigested components, including NSP in maize-based diets, and boost growth rate and production efficiency in broilers.

Maize contains a lower level of soluble NSP compared to wheat, but both maize and wheat have similar levels of insoluble NSP (Choct, 2006; Bach Knudsen, 2014). Diets containing 60% to 70% maize have about half the soluble NSP content of a standard wheat-based diet (Kim et al., 2022) and do not create a viscous gut environment to a level that negatively impacts nutrient absorption in poultry (Bach Knudsen, 2014). However, maize-based diets still contain substantial amounts of insoluble NSP (Kim et al., 2022), which can reduce the nutritional value of feed by trapping nutrients within the cell wall matrix and creating a physical barrier during digestion (Hetland et al., 2004; Choct, 2015). Therefore, supplementation of NSP-degrading enzymes is required in maize-based diets to improve the nutritive value of feed. Gilani et al. (2021) observed increases in final body weight, average daily gain, and a tendency toward improved feed conversion ratio (FCR) in broilers fed maize-based diets supplemented with a xylanase and β-glucanase preparation (XG). Soybean meal, the major protein source in poultry diets, contains between 20% and 30% NSP, of which 8% is cellulose and the remaining is pectic polysaccharides and oligosaccharides (Choct, 1997; Choct et al., 2010). In this regard, the supplementation of multiple NSP-degrading enzymes may aid NSP degradation by targeting various NSP present in diets simultaneously.

Soybean meal contains 1% to 2% mannose (Bach Knudsen, 1997), which is the building block of mannans. A recent study by Rueckel et al. (2024), using a novel visualisation concept, showed that β-mannanase was active not only at the cell wall and hull of soybean meal but also within the intracellular components. Based on this, the authors hypothesised that mannans or their precursors might be present in the cotyledons of soybeans. However, the exact form or concentration of mannans in soybean meal remains unknown. Early research by Whistler and Saarnio (1957) identified the presence of galactomannan in soy hulls, with a D-galactose to D-mannose ratio of 2:3. These mannans can be targeted and degraded by β-mannanase (Shastak et al., 2015). In previous study (Kim et al., 2025), β-mannanase supplementation in a wheat-based broiler diet led to six points of improvement in FCR and a 2% increase in weight gain at 35 d of age through increased NSP utilization and an improved gut environment. The study also demonstrated that the combination of β-mannanase and XG further enhanced energy utilization and butyric acid production in broilers. These synergistic effects of β-mannanase and XG in wheat-based diets observed in the above study were significant and necessitate further investigation in maize-based diets. While the performance benefits of β-mannanase or XG supplementation in maize-based diets have been reported (Jackson et al., 2004; Li et al., 2010; Cowieson et al., 2010; Kiarie et al., 2021), most of these studies excluded phytase from the basal diet, limiting the understanding of the added value of β-mannanase or XG in commercial broiler diets. The presence of phytase in basal diets likely masks the growth performance benefits stemming from β-mannanase or XG supplementation due to the beneficial effects of phytase on nutrient and energy digestibility (Selle and Ravindran, 2007). However, the combined effects of β-mannanase and XG in maize-based diets containing phytase have not been studied in broilers. Given that dietary mannans can be degraded by mannanase and XG can hydrolyze arabinoxylans and β-glucans in feed, it is plausible that a strategic enzyme blend of XG and β-mannanase could more effectively hydrolyze the diverse NSP present in maize-based feed. This study evaluated the effects of β-mannanase, alone or in combination with XG, on growth performance, nutrient and energy utilization, NSP degradation, and the gastrointestinal environment of broilers fed maize-based diets.

## 2 Materials and methods

### 2.1 Animal ethics statement

This experiment was conducted with the approval of the Animal Ethics Committee of the University of New England (UNE), Australia (approval No. ARA22-043). All broiler management procedures, including health care, husbandry, and animal use, complied with the requirements of the Australian Code for the Care and Use of Animals for Scientific Purposes (NHMRC, 2013).

### 2.2 Housing and bird management

A total of 384 mixed-sex Cobb 500 broiler chicks were obtained from the Baiada hatchery in Tamworth, New South Wales, Australia. On hatch day (d 0), the birds were randomly assigned to 32 equally sized floor pens (12 birds/pen) in the environment-controlled Rob Cumming Poultry Innovation Centre at UNE. Each pen was equipped with a feeder and two nipple drinkers, and birds had ad libitum access to feed and water. Hardwood shavings were spread to a depth of approximately 7 cm in each pen. The room temperature was initially set at 33 °C and gradually reduced to 22 °C by d 21, where it remained until d 35. The lighting regimen provided 23 h of light at approximately 40 lux on d 1, with darkness increasing by 1 h per day until reaching 6 h of darkness, after which 18 h of light per day at 10 lux was maintained for the remainder of the study.

### 2.3 Diets and experimental design

The diets were thoroughly mixed and cold pelleted at 65 °C, with the starter diets crumbled. The basal diet contained maize and soybean meal as major ingredients and were analyzed for crude protein (CP) and amino acids by near-infrared spectroscopy (NIR Systems 6500, Foss, Hillerød, Denmark) standardized to Evonik AMINONIR advanced calibration before formulation. The ingredients and nutrient levels of the basal diet are presented in Table 1. All dietary treatments contained phytase (Natuphos E 10000 G, BASF SE Animal Nutrition, Ludwigshafen, Germany) at 1000 FTU/kg, with matrix values assigned for energy, amino acids, and minerals. The diets were formulated to meet Cobb 500 nutrient specifications (Cobb-Vantress, 2022). Vitamin and mineral premixes were included according to the manufacturer's recommendations (Rabar Pty Ltd., Beaudesert, Queensland, Australia). Titanium dioxide was added as an indigestible marker at 0.5% in the grower diet. Birds received a starter crumble for the first 10 days post–hatch, followed by 3 mm pelleted grower and finisher diets from d 11 to 21 and d 22 to 35, respectively.

**Table 1:** Ingredients and nutrient levels of the basal diet (dry matter basis, %).

Item	Starter (d 0–10)	Grower (d 11–21)	Finisher (d 22–35)
**Ingredients**			
Maize	54.92	56.84	59.03
Wheat bran	5.00	7.50	10.00
Soybean meal	33.6	29.7	25.80
Canola oil	3.00	2.80	2.70
DL-Methionine	0.22	0.15	0.16
L-Threonine	0.03	0.01	0.00
Choline chloride 60%	0.11	0.11	0.11
Vitamin premix^1^	0.09	0.09	0.09
Mineral premix^2^	0.11	0.11	0.11
Sand	0.42	0.42	0.42
Titanium dioxide	0.00	0.50	0.00
Limestone	0.81	1.08	1.03
Salt	0.30	0.31	0.32
Dicalcium phosphate	1.38	0.37	0.22
Phytase^3^	0.01	0.01	0.01
Total	100.00	100.00	100.00
**Calculated values**			
Dry matter	89.4	89.3	89.1
Crude protein	21.2	20.1	18.7
AME_n_, kcal/kg	3084	3080	3094
Dig Lys	1.13	1.04	0.95
Dig Met	0.53	0.44	0.44
Dig Met + Cys	0.85	0.75	0.74
Dig Thr	0.77	0.70	0.65
Dig Val	1.00	0.94	0.88
Dig Iso	0.90	0.84	0.78
Dig Arg	1.44	1.34	1.23
Dig Trp	0.25	0.23	0.22
Ca	0.96	0.80	0.74
Available P	0.48	0.40	0.37
Na	0.16	0.16	0.16
Choline, mg/kg	1700	1600	1550
**Analysed values**			
Gross energy, kcal/kg	3932	3934	3958
Crude protein	20.1	19.8	18.5
Ca	1.49	1.09	1.12
P	1.36	0.93	0.95
Soluble NSP	–	0.78	–
Insoluble NSP	–	7.85	–
Total NSP	–	8.63	–
Free oligosaccharides	–	4.78	–

AME_n_ = apparent metabolizable energy corrected for N retention; Dig = digestible; NSP = non-starch polysaccharides.

1 Vitamin premix per kg diet (Rabar Pty Ltd. Beaudesert, Queensland, Australia): vitamin A, 12 MIU; vitamin D, 5 MIU; vitamin E, 75 mg; vitamin K, 3 mg; nicotinic acid, 55 mg; pantothenic acid, 13 mg; folic acid, 2 mg; riboflavin, 8 mg; cyanocobalamin, 0.016 mg; biotin, 0.25 mg; pyridoxine, 5 mg; thiamine, 3 mg; antioxidant, 50 mg.

2 Mineral premix per kg diet (Rabar Pty Ltd. Beaudesert, Queensland, Australia): Cu, 16 mg as copper sulfate; Mn, 60 mg as manganese sulfate; Mn, 60 mg as manganous oxide; I, 0.125 mg as potassium iodide; Se, 0.3 mg; Fe, 40 mg, as iron sulfate; Zn, 50 mg as zinc oxide; Zn, 50 mg as zinc sulfate.

3 Natuphos E 10000 G (BASF SE Animal Nutrition, Ludwigshafen, Germany).

All birds were weighed upon arrival and assigned to four treatments in a 2 × 2 factorial arrangement, with β-mannanase (with or without) and XG (with or without) as factors. The treatments included: 1) a basal diet with phytase at 1000 FTU/kg (unsupplemented control), and the basal diet supplemented with 2) β-mannanase (Natupulse TS, BASF SE Animal Nutrition, Ludwigshafen, Germany; 800 TMU/kg), 3) XG (Natugrain TS, BASF SE Animal Nutrition, Ludwigshafen, Germany; 560 TXU/kg of xylanase and 250 TGU/kg XG), and 4) a combination of XG (560 TXU/kg of xylanase and 250 TGU/kg of XG) and β-mannanase (800 TMU/kg). Each treatment included eight replicate pens of 12 birds each, totalling 96 birds per treatment. One thermostable endo-xylanase unit (TXU) is defined as the amount of enzyme that releases 5 μmol of reducing sugars, measured as xylose equivalents per minute from a buffer solution containing 1 g of arabinoxylan per 100 mL at pH 3.5 and 40 °C. One thermostable endo-glucanase unit (TGU) is defined as the amount of enzyme that releases 1 μmol of reducing sugars, measured as glucose equivalents per minute from a buffer solution containing 0.714 g β-glucan per 100 mL at pH 3.5 and 40 °C. One thermostable mannanase unit (TMU) is defined as the amount of enzyme that produces reducing carbohydrates having a reducing power corresponding to one micromole mannose from locust bean gum (0.3 g/100 mL buffer solution) in 1 min under the assay conditions of 50.0 ± 0.1 °C and pH 3.5. The feed supplement was added at the expense of sand.

### 2.4 Growth performance

Birds and feed were weighed upon arrival and on d 10, 21, and 35 on a pen basis. Mortality was recorded daily. The feed intake, weight gain, and mortality-adjusted FCR were calculated for each growth stage.

### 2.5 Apparent total tract retention of nutrients and metabolizable energy

On d 15, four birds per pen (six replicates/treatment) were transferred to cages and acclimatized for two days. Fresh water and feed were provided ad libitum. From d 17 to 20, feed intake and total excreta output were recorded daily for three consecutive days to determine apparent metabolizable energy (AME) and nitrogen-corrected AME (AME_n_), apparent total tract retentions of dry matter (DM), nitrogen, calcium, phosphorus, and apparent total tract degradations of soluble NSP, insoluble NSP, and free oligosaccharides. At the end of the collection period, multiple sub-samples of excreta were collected, homogenized, and a 250-g representative sample was stored in a plastic container at −20 °C until further processing.

### 2.6 Apparent ileal digestibility of nutrients, intestinal viscosity, and pH

On d 21, caged birds were euthanized by electrical stunning (MEFE CAT 44N, Mitchell Engineering Food Equipment, Clontarf, Queensland, Australia) followed by cervical dislocation. The carcass was dissected to measure gut pH and collect gut contents for assessing viscosity, apparent ileal digestibility of DM and CP, and caecal short-chain fatty acids (SCFA). The jejunal, ileal, and caecal contents were pooled per cage. The pH of the gizzard, jejunum, and ileal contents were measured in situ using a pH probe (Sensorex, Garden Grove, CA, USA) connected to a pH meter (Mettler-Toledo Ltd., Leicester, UK). The probe was inserted directly into the digesta without touching the intestinal wall. Each measurement was taken twice, and the average pH value was recorded. The probe was rinsed with ultra-pure water (ICW 3000 water purifier for ion chromatography, Millipore, Burlington, MA, USA) between samples. Ileal digesta viscosity was measured in triplicate for each cage. Ileal digesta samples were transferred into 2 mL Eppendorf tubes and centrifuged at 10,000 × *g* for 10 min at room temperature. Viscosity was measured using 0.5 mL of the resulting supernatant with a Brookfield DV3T Rheometer (Brookfield Ametek, Instrumentation & Specialty Controls Division, Middleboro, MA, USA), with CPA-40Z Spindle, at 25 °C. Viscosity measurements were expressed in centipoise (cPs) unit (1 cPs = 1/100 dyne·s/cm^2^ = 1 mPa·s) before statistical analysis. The remaining ileal and caecal contents were stored at −20 °C until further processing.

### 2.7 Caecal SCFA

The SCFA and lactic acid concentrations in caecal digesta samples were measured using a modified version of the method described by Jensen et al. (1995). Briefly, 1 mL of internal standard (0.01 mol/L ethylbutyric acid) was added to approximately 2 g of fresh digesta, mixed, and centrifuged at 3900 × *g* at 5 °C for 20 min. Approximately 1 mL of the resulting supernatant was combined with 0.5 mL of concentrated HCl (36%) and 2.5 mL of ether. The mixture was centrifuged at 2000 × *g* at 5 °C for 15 min. Then, 400 μL of the resulting supernatant was mixed with 40 mL of N-tert-butyldi-methlsilyl-N-methyltrifuoroacetamide (MTBSTFA) in a gas chromatography (GC) vial. The samples were heated at 80 °C for 20 min, left at room temperature for 48 h, and analyzed using a Varian CP3400 CX gas chromatograph (Varian Analytical Instruments, Palo Alto, CA, USA). The SCFA concentration was expressed as μmol/g of digesta.

### 2.8 Measurements and chemical analyses

Frozen excreta and ileal digesta samples were dried using a freeze-drier (Alpha 1-4 LDplus, Marin Christ, Osterode am Harz, Lower Saxony, Germany) and ground to pass through a 0.5 mm screen. Ground feed, excreta, and ileal contents were analyzed in duplicate for nitrogen using the Dumas method with a LECO FP-2000 automatic nitrogen analyzer (Leco Corporation, St. Joseph, MI, USA). The analysed nitrogen values were corrected for CP by multiplying the correction factor of 6.25. Titanium dioxide in diet and digesta samples was measured in duplicate by UV-spectroscopy at 410 nm (Cary 50 Bio UV–Visible spectrophotometer equipped with a Cary 50 MPR microplate reader, Varian Inc., Palo Alto, CA, USA) following the method described by Short et al. (1996). The DM content of ground feed and freeze-dried samples was determined using oven-drying following the method 934.01 (AOAC, 1990). The gross energy in feed and excreta was measured using a 0.5 g sample in an adiabatic bomb calorimeter (IKA Werke, C7000, GMBH and Co., Staufen, Germany) with benzoic acid as the standard. The X-ray fluorescence spectroscopy analysis was conducted using the Olympus Vanta M Series (Olympus Scientific Solutions Americas, Waltham, MA, USA) to detect calcium and phosphorus in the feed and excreta samples, with 1 g of sample placed in polyethylene XRF sample cups. The standard scan method used was the Geochem 3-beam method with 30 s scan per beam and 90 s in total (Vanta User's Manual, 2025). The composition of soluble and insoluble NSP and free oligosaccharides in feed and excreta samples (d 21) was determined using an Agilent 8890 GC equipped with an Agilent 7693A autosampler (Agilent Technologies Inc., Palo Alto, CA, USA). The analysis followed the procedure of Englyst et al. (1994) with modifications as described by Theander et al. (1995) and Morgan et al. (2019).

### 2.9 Calculations

The AME, apparent total tract retention, and apparent ileal digestibility of nutrients were calculated by using the following equations:

$$\text{AME}=\frac{\left(\text{Feed}\;\text{intake}\times \text{GE}\;\text{diet}\right)-\left(\text{Excreta}\;\text{output}\times \text{GE}\;\text{excreta}\right)}{\text{Feed}\;\text{intake}}\text{.}$$

AME_n_ was determined by correction for zero nitrogen retention by simple multiplication with 8.22 kcal/g nitrogen retained in the body.

$$\text{Apparent}\;\text{total}\;\text{tract}\;\text{retention}\;\text{of}\;\text{nutrient}\;\left(\%\right)=\frac{\left(\text{Feed}\;\text{intake}\times \text{Nutrient}\;\text{in}\;\text{diet}\right)-\left(\text{Excreta}\;\text{output}\times \text{Nutrient}\;\text{in}\;\text{excreta}\right)}{\left(\text{Feed}\;\text{intake}\times \text{Nutrient}\;\text{in}\;\text{diet}\right)}\times 100\text{.}$$

Apparent ileal digestibility of nutrient (%) = (Ti diet/Ti digesta) × (Nutrient in digesta/Nutrient in diet) × 100.

### 2.10 Statistical analysis

Data were analyzed using the General Linear Model procedures in IBM SPSS statistical software version 28.0. The pen or the cage was considered the experimental unit for statistical analysis. Normality was assessed using the Kolmogorov–Smirnov test. A two-way ANOVA was performed to evaluate the main effects of XG, β-mannanase, and their interaction. The statistical model used was as follows:

$$Y_{ij}=\mu +M_{i}+X_{j}+R_{ij}+\varepsilon_{ij}\text{,}$$

where *Y_ij_* is the observation of dependent variables; *μ* is the overall mean; *M_i_* is the β-mannanase effect; *X_j_* is the XG effect, *R_ij_* represents the interactive effect between the two factors, and *ε_ij_* is the residual error for the observation.

The sex ratio of each pen was applied as a covariate in the analysis. When significant differences were detected, means were separated using Tukey's HSD test. Non-normally distributed data were analysed using the Kruskal–Wallis test. Statistical significance was set at *P <* 0.05, while trends towards significance were considered at 0.05 ≤ *P* ≤ 0.10.

## 3 Results

### 3.1 Growth performance

The effect of in-feed carbohydrases on the growth performance of broilers is presented in Table 2. During d 0 to 10, dietary supplementation of β-mannanase increased weight gain by 4.7% (*P* = 0.009) and improved FCR by 5 points (*P* < 0.001) compared to the unsupplemented control, while XG supplementation had no effects on both weight gain and FCR (*P* > 0.05). During d 11 to 21, β-mannanase supplementation improved FCR by six points (*P* = 0.039), and β-mannanase and XG interacted for weight gain (*P* = 0.008) where β-mannanase or XG alone increased weight gain compared to the unsupplemented control but there were no further improvements achieved via their simultaneous use. During d 22 to 35, XG supplementation increased weight gain by 4.6% (*P* = 0.003) and improved FCR by 6 points (*P* = 0.032), while β-mannanase had no effects on both weight gain and FCR (*P* > 0.05). During d 0 to 35, XG supplementation improved FCR (*P* = 0.019) but XG and β-mannanase tended to interact for FCR (*P* = 0.071) with either of the enzymes improving FCR compared to the unsupplemented control but with no further improvements when added in tandem. Dietary XG and β-mannanase interacted for weight gain (*P* = 0.026) where XG improved weight gain in the absence of β-mannanase but there were no further improvements achieved via their simultaneous use. The dietary supplementation of XG or β-mannanase had no effect on feed intake during any of the feeding phases (*P >* 0.05). Mortality was <3% and not affected by dietary treatment (data not shown).

**Table 2:** Effects of in-feed carbohydrases on growth performance of broilers.

Item		Weight gain, g/bird				Feed intake, g/bird				Feed conversion ratio, g/g			
		d 0 to 10	d 11 to 21	d 22 to 35	d 0 to 35	d 0 to 10	d 11 to 21	d 22 to 35	d 0 to 35	d 0 to 10	d 11 to 21	d 22 to 35	d 0 to 35
**Treatment**^1^													
XG	β-Mannanase												
–	–	253	684^b^	1293	2230^c^	306	992	1989	3286	1.208	1.449	1.542	1.476
–	+	265	715^a^	1305	2285^bc^	306	957	2010	3273	1.156	1.335	1.542	1.433
+	–	254	733^a^	1379	2366^a^	305	1005	1982	3292	1.203	1.373	1.440	1.391
+	+	267	725^a^	1339	2331^ab^	306	982	2045	3333	1.142	1.354	1.527	1.428
SEM		4.4	9.8	19.5	19.1	4.7	25.4	33.3	41.0	0.0107	0.0303	0.0272	0.0188
**Main effect**													
XG	–	259	700	1299	2257	306	974	2000	3280	1.182	1.392	1.542	1.455
	+	261	729	1359	2349	305	993	2014	3313	1.173	1.363	1.483	1.410
β-Mannanase	–	254	709	1336	2298	305	998	1986	3289	1.205	1.411	1.491	1.434
	+	266	720	1322	2308	306	969	2028	3303	1.149	1.345	1.534	1.431
***P*-value**													
XG		0.651	0.004	0.003	<0.001	0.926	0.431	0.662	0.713	0.400	0.330	0.032	0.019
β-Mannanase		0.009	0.246	0.475	0.611	0.925	0.261	0.215	0.107	<0.001	0.039	0.123	0.880
XG × β-mannanase		0.892	0.008	0.053	0.026	0.965	0.817	0.495	0.434	0.675	0.124	0.115	0.071

XG = xylanase and β-glucanase preparation; SEM = standard error of the mean.

Means within columns not sharing a common superscript are significantly different (*P* < 0.05). *n* = 8.

1 The treatments included: 1) a basal diet with phytase at 1000 FTU/kg (unsupplemented control), and the basal diet supplemented with 2) β-mannanase (800 TMU/kg), 3) XG (560 TXU/kg of xylanase and 250 TGU/kg of xylanase and β-glucanase, respectively), and 4) a combination of XG (560 TXU/kg of xylanase and 250 TGU/kg of β-glucanase) and β-mannanase (800 TMU/kg).

### 3.2 Intestinal viscosity and pH

The effects of in-feed carbohydrases on intestinal viscosity and pH of broilers on d 21 are presented in Table 3. Dietary supplementation of XG or β-mannanase had no effect on jejunal and ileal viscosity (*P >* 0.05). Neither of the enzymes had any effect on the pH of the gizzard, jejunum, and ileum contents (*P >* 0.05).

**Table 3:** Effects of in-feed carbohydrases on intestinal viscosity and pH of broilers on d 21.

Item		Viscosity, mPa·s		pH		
		Jejunum	Ileum	Gizzard	Jejunum	Ileum
**Treatment**^1^						
XG	β-Mannanase					
–	–	1.93	2.47	2.64	5.66	6.22
–	+	2.06	2.81	2.60	6.05	6.25
+	–	1.97	3.02	2.68	6.01	6.60
+	+	2.05	2.80	2.45	5.73	6.41
SEM		0.058	0.160	0.102	0.184	0.256
**Main effect**						
XG	–	1.99	2.64	2.62	5.87	6.24
	+	2.01	2.91	2.57	5.87	6.50
β-Mannanase	–	1.95	2.74	2.66	5.85	6.41
	+	2.06	2.80	2.53	5.89	6.33
***P*-value**						
XG		0.820	0.112	0.622	0.979	0.309
β-Mannanase		0.071	0.720	0.207	0.851	0.755
XG × β-mannanase		0.629	0.102	0.373	0.102	0.679

XG = xylanase and β-glucanase preparation; SEM = standard error of the mean.

Means within columns not sharing a common superscript are significantly different (*P* < 0.05). *n* = 8.

1 The treatments included: 1) a basal diet with phytase at 1000 FTU/kg (unsupplemented control), and the basal diet supplemented with 2) β-mannanase (800 TMU/kg), 3) XG (560 TXU/kg of xylanase and 250 TGU/kg of xylanase and β-glucanase, respectively), and 4) a combination of XG (560 TXU/kg of xylanase and 250 TGU/kg of β-glucanase) and β-mannanase (800 TMU/kg).

### 3.3 Apparent ileal digestibility and total tract retention of nutrients and AME

The effects of in-feed carbohydrases on apparent ileal digestibility, total tract retention of nutrients, and metabolizable energy of broilers are presented in Table 4. There was no effect of XG or β-mannanase supplementation on the apparent ileal digestibility of DM (*P >* 0.05). Dietary XG supplementation tended to increase the apparent ileal digestibility of N (*P* = 0.069). Dietary β-mannanase supplementation increased the apparent total tract retentions of DM (*P* = 0.001), calcium (*P* = 0.020), AME (*P* < 0.001), AME_n_ (*P* < 0.001), and increased apparent total tract degradations of insoluble NSP (*P* = 0.010) and total NSP (*P* = 0.010). Dietary β-mannanase supplementation tended to increase the total tract degradation of soluble NSP (*P* = 0.104) but had no effect on the total tract degradation of free oligosaccharides and retention of phosphorus (*P* > 0.05). Dietary β-mannanase and XG interacted for total tract retention of N (*P* = 0.002), where, compared to the unsupplemented control, β-mannanase increased total tract N retention of birds in the absence of XG but not in the presence of XG. XG supplementation had no effect on apparent total tract retentions of DM, calcium, phosphorus, AME, AME_n_, and total tract degradations of soluble NSP, insoluble NSP, total NSP, and free oligosaccharides (*P* > 0.05).

**Table 4:** Effects of in-feed carbohydrases on apparent ileal digestibility, total tract retention of nutrients, and metabolizable energy of broilers (DM basis).

Item		Apparent ileal digestibility, %		Apparent total tract retention, %								AME, kcal/kg	AMEn, kcal/kg
		DM	N	DM	N	Soluble NSP	Insoluble NSP	Total NSP	Free oligo saccharides	Ca	P		
**Treatment**^1^													
XG	β-Mannanase												
–	–	68.4	82.3	75.0	60.6^b^	49.7	24.4	26.7	82.6	72.8	69.5	3425	3331
–	+	68.3	82.9	77.3	66.3^a^	56.0	31.8	34.1	83.7	76.2	70.0	3555	3427
+	–	68.3	84.0	75.2	61.9^b^	48.9	22.9	25.4	85.2	73.4	70.2	3443	3341
+	+	69.0	83.3	76.9	62.5^b^	56.9	36.4	38.2	85.2	77.5	66.6	3537	3445
SEM		0.56	0.54	0.55	0.73	4.19	3.63	3.54	1.54	1.49	2.22	21.4	19.1
**Main effect**													
XG	–	68.4	82.6	76.1	63.4	52.9	28.1	30.4	83.2	74.5	69.8	3490	3379
	+	68.6	83.6	76.0	62.2	52.9	29.7	31.8	85.2	75.4	68.4	3490	3393
β-Mannanase	–	68.3	83.2	75.0	61.3	49.3	23.7	26.1	83.9	73.1	69.9	3434	3336
	+	68.7	83.1	77.1	64.4	56.5	34.1	36.2	84.5	76.9	68.3	3546	3436
***P*-value**													
XG		0.670	0.069	0.824	0.115	0.992	0.668	0.694	0.198	0.546	0.541	0.995	0.480
β-Mannanase		0.587	0.903	0.001	<0.001	0.104	0.010	0.010	0.721	0.020	0.486	<0.001	<0.001
XG × β-mannanase		0.491	0.208	0.625	0.002	0.845	0.410	0.458	0.714	0.805	0.370	0.414	0.842

XG = xylanase and β-glucanase preparation; DM = dry matter; N = nitrogen; NSP = non-starch polysaccharides; AME = apparent metabolizable energy; AME_n_ = apparent metabolizable energy corrected for nitrogen; SEM = standard error of the mean.

Means within columns not sharing a common superscript are significantly different (*P* < 0.05). *n* = 8.

1 The treatments included: 1) a basal diet with phytase at 1000 FTU/kg (unsupplemented control), and the basal diet supplemented with 2) β-mannanase (800 TMU/kg), 3) XG (560 TXU/kg of xylanase and 250 TGU/kg of xylanase and β-glucanase, respectively), and 4) a combination of XG (560 TXU/kg of xylanase and 250 TGU/kg of β-glucanase) and β-mannanase (800 TMU/kg).

### 3.4 Caecal SCFA

The effect of in-feed carbohydrases on the caecal SCFA profile of broilers on d 21 is presented in Table 5. Dietary β-mannanase and XG interacted for acetic acid (*P* = 0.034) and total SCFA (*P* = 0.044) concentrations in the caecal contents, where β-mannanase alone increased the concentration of acetic acid and total SCFA compared to the unsupplemented control but there were no further improvements (*P >* 0.05) when both the enzymes were added in tandem. There was no effect of XG or β-mannanase on the concentrations of formic acid, propionic acid, butyric acid, isobutyric acid, valeric acid, isovaleric acid, and lactic acid in the caeca (*P >* 0.05).

**Table 5:** Effects of in-feed carbohydrases on caecal short-chain fatty acid (SCFA) profile (μmol/g) of broilers on d 21.

Item		Formic acid	Acetic acid	Propinoic acid	Butyric acid	Isobutyric acid	Valeric acid	Isovaleric acid	Total SCFA	Lactic acid
**Treatment**^1^										
XG	β-Mannanase									
–	–	0.28	51.8^b^	4.7	16.5	0.60	0.87	0.16	74.9^b^	0.18
–	+	0.25	76.0^a^	4.0	17.8	0.67	0.78	0.18	99.7^a^	0.74
+	–	0.29	65.2^ab^	3.9	19.1	0.65	0.86	0.20	90.2^ab^	0.12
+	+	0.23	65.0^ab^	4.6	17.4	0.77	0.88	0.22	89.1^ab^	0.18
SEM		0.045	5.38	0.34	1.10	0.067	0.042	0.031	6.02	0.314
**Main effect**										
XG	–	0.26	63.9	4.4	17.2	0.63	0.82	0.72	87.3	0.46
	+	0.26	65.1	4.2	18.3	0.71	0.87	0.21	89.6	0.15
β-Mannanase	–	0.28	58.5	4.3	17.8	0.63	0.86	0.18	82.5	0.15
	+	0.24	70.5	4.3	17.6	0.72	0.83	0.20	94.4	0.46
***P-*value**										
XG		0.927	0.820	0.730	0.499	0.283	0.462	0.207	0.698	0.334
β-Mannanase		0.322	0.037	0.961	0.891	0.199	0.567	0.593	0.064	0.339
XG × β-mannanase		0.649	0.034	0.142	0.349	0.705	0.330	0.957	0.044	0.433

XG = xylanase and β-glucanase preparation; SEM = standard error of the mean.

Means within columns not sharing a common superscript are significantly different (*P* < 0.05). *n* = 8.

1 The treatments included: 1) a basal diet with phytase at 1000 FTU/kg (unsupplemented control), and the basal diet supplemented with 2) β-mannanase (800 TMU/kg), 3) XG (560 TXU/kg of xylanase and 250 TGU/kg of xylanase and β-glucanase, respectively), and 4) a combination of XG (560 TXU/kg of xylanase and 250 TGU/kg of β-glucanase) and β-mannanase (800 TMU/kg).

## 4 Discussion

In poultry, a common strategy to enhance the nutritive value of feed ingredients, improve performance, reduce nutrient excretion, and maximize return on investment is to supplement diets with exogenous enzymes. Particularly, poultry do not efficiently digest or utilise NSP due to their lack of endogenous enzymes for NSP hydrolysis. Soluble NSP has been problematic due to their antinutritive effects, such as increasing digesta viscosity that hinders nutrient utilisation. Thus, the use of phytase and carbohydrases is now a common practice during the feed formulation; however, the selection of enzymes must be carefully considered due to the complex chemical structure of NSP present in complete diets. A single enzyme activity may not effectively hydrolyse NSP present in poultry feed, and tailoring enzymes to the substrates available in complete feed is key to maximise the efficacy of enzymes. Xylanase with or without XG has been extensively explored in broilers fed viscous grains such as wheat and barley. In this study, β-mannanase was used either alone or in combination with XG to improve growth performance, as well as the utilization of nutrients, energy, and NSP in broilers fed maize-based diets containing phytase.

Broilers fed the basal diet (unsupplemented control) presented 75% total tract DM utilisation measured by total excreta collection in the present study at 21 d of age, which is similar to previous reports (Olukosi et al., 2008; Stefanello et al., 2016). This is higher than the 68% apparent total tract DM digestibility measured using TiO_2_ as a marker reported by Kim et al. (2022) where phytase was not used. This likely suggests that the phytase in the basal diet improved nutrient utilization of broilers, irrespective of supplemental carbohydrases, in the present study. The beneficial effects of phytase on nutrient utilization and growth performance are well documented (Selle and Ravindran, 2007) and will not be discussed here. However, it is important to note that any performance improvements in broilers observed from the supplemental carbohydrases in the present study are in addition to the effects of phytase in the unsupplemented control. While supplementation of XG did not affect the total tract retention of nutrients from d 17 to 20, β-mannanase improved it.

The present study observed the improvements in growth performance mediated by positive effects of β-mannanase during the starter and grower phases (d 0 to 10 and d 11 to 21), though no significant effects were observed during the finisher period (d 22 to 35). During the starter and grower phases, β-mannanase improved both weight gain and FCR. These findings align with previous studies (Kong et al., 2011; Latham et al., 2018; Macelline et al., 2023), collectively suggesting that antinutritive effects of dietary mannans or other mannooligomer-based antigens are likely more pronounced in younger birds. As birds age, their digestive system may better adapt to the dietary components and become less sensitive to the antinutrients. The starter diets contained higher levels of soybean meal (SBM) compared to the finisher diets, and therefore more antinutrients, which may explain the more pronounced responses of young birds to β-mannanase supplementation. In contrast, other studies with maize-based diets have reported improvements in growth performance from β-mannanase supplementation regardless of bird age (Jackson et al., 2004; Zou et al., 2006; Kiarie et al., 2021). The absence of phytase and high levels of mannans or mannose-containing antigens in the basal diet likely resulted in increased response to β-mannanase in the above studies. Maize-based diets contain about half the soluble NSP content of a standard wheat-based diet (Kim et al., 2022), and a significant increase in intestinal viscosity is not expected by feeding these diets to poultry. The viscosity of the intestinal contents of birds in the current study was already lower than those fed a wheat-based diet with supplemental XG in previous study (Kim et al., 2025). For this reason, further reductions in the intestinal viscosity were not achieved by the addition of either β-mannanase or XG in the current study. The improved growth performance by β-mannanase supplementation was associated with improved utilization of NSP, N, minerals and AME and AME_n_. Some of these benefits were also observed in previous studies where β-mannanase was supplemented in broiler diets based on wheat (Barekatain et al., 2024; Kim et al., 2025, submitted) and corn (Ferreira et al., 2016; Sacakli et al., 2024). Notably, β-mannanase increased AME and AME_n_ by 3.3% and 3.0%, respectively, translating to an additional approximately 100 kcal/kg of dietary energy, an improvement of significant nutritional and economic values. These improvements partially resulted from enhanced insoluble NSP degradation. This degradation or partial solubilization of insoluble NSP by supplemental β-mannanase may have alleviated the “cage effects” of insoluble fibre, releasing nutrients trapped within the plant cell wall matrix and making them more available to birds. Increased total tract retention of DM and Ca may have resulted from such effects of β-mannanase. Another possible explanation is the synergistic effects of β-mannanase and phytase. Either enzyme may have disrupted plant cell wall structures or loosened the phytate complex embedded in cell walls, thereby improving the accessibility of other enzymes to target substrates.

The degradation of insoluble and total NSP with β-mannanase supplementation observed in the present study suggests successful hydrolysis of dietary fibre. As a result, more readily fermentable oligosaccharides are released within the gut, which have prebiotic effects (Shastak et al., 2015). This was evident in the present study where caecal fermentation was improved by supplemental enzymes with increased acetic acid and total SCFA concentrations in the caeca, compared to non-supplemented birds. Kalidas et al. (2017) isolated MOS from palm kernel cake and reported a significant increase in the growth of the beneficial *Lactobacillus reuteri* C1 strain in vitro. Growing evidence suggests that β-mannanase can positively modulate gut health, immune response, and microbiota population in broilers (Bortoluzzi et al., 2019; Barekatain et al., 2024; Zhang et al., 2024). These effects may be linked to enhanced fibre fermentation by caecal bacteria as observed in the current study. The quantification of MOS released in situ followed by the enzymatic hydrolysis of dietary mannans, along with their impacts on gut microbiota abundance and diversity, should be investigated in future studies.

Interestingly, in the present study, XG supplementation had no effect on growth performance during the first 10 days (starter phase) but progressively improved performance from the grower phase, with the maximum response observed during the finisher phase. This corroborates the findings of De Keyser et al. (2018) and Gilani et al. (2021), who observed improved body weight and FCR during the grower and finisher phases but not in the starter phase by XG supplementation. In contrast, Pasquali et al. (2021) reported an increased body weight of 6.8% during the first 7 days but progressively reduced in the grower phase with no response in the finisher phase, highlighting a more pronounced response of XG in younger birds which is the opposite to what was observed in the present study. One explanation of the lack of effects of supplemental XG on growth performance in the starter phase is that the basal starter diet contained sufficient AME (approximately 3100 kcal/kg) and 1000 FTU/kg phytase, and the capacity for improvement in weight gain or feed efficiency by XG was marginal (Adeola and Cowieson, 2011), especially with the lower presence of substrates that can be targeted by XG in maize-based diets. Hence, all birds performed above-breed performance objectives during the starter period, which may limit further improvement by supplemental enzymes. However, it should be noted that the inclusion levels of wheat bran gradually increased from the starter to finisher phases (5% to 10%), thereby increasing the dietary levels of arabinoxylans for XG. It appears that supplementing XG released fermentable oligosaccharides via the hydrolysis of long-chain polysaccharides in wheat bran which can promote the proliferation of fibre-fermenting bacteria from a young age. This may enable gut microbiota to more effectively degrade the highly complex NSP later in life, i.e., during the grower and even more in the finisher phase (Bedford, 2025). Since the basal diet contained phytase, some degree of improvement in nutrient utilization may have already been achieved, thereby reducing the undigested fraction available as substrates for XG to act upon. Supplemental XG, however, tended to increase the apparent ileal digestibility of nitrogen, possibly by degrading cell wall structures to release proteins from the cells and exposing them to endogenous enzymes in the digestive tract (Cowieson et al., 2010). Although the effect of XG on caecal SCFA production was not clear, the interaction between XG and β-mannanase on caecal acetic acid and SCFA concentrations suggests some in situ production of AXOS, leading to increased caecal fermentation capacity. The mechanism of action of XG during the grower-finisher period would have been clearer if measurements of nutrient digestibility, NSP degradation, and caecal SCFA concentrations had been conducted at 35 d of age, as pronounced effects of XG were observed in the later stages of growth.

## 5 Conclusion

This experiment demonstrated that β-mannanase supplementation improved growth performance in the early stages of broiler development, likely through enhancing nutrient and energy utilization when supplemented to the maize-based diet. Notably, β-mannanase also led to greater caeca SCFA production, indicating successful fibre degradation and fermentation. On the other hand, XG improved growth performance during the grower-finisher period. However, the combination of XG and β-mannanase did not result in synergistic effects on growth performance, nutrient, and energy utilization in broilers, possibly due to limited room for improvement under the conditions of the present study.

## CRediT authorship contribution statement

**Eunjoo Kim:** Writing – review & editing, Methodology, Investigation, Formal analysis, Data curation. **Mingan Choct:** Writing – review & editing, Supervision, Investigation, Funding acquisition, Conceptualization. **Anna Fickler:** Writing – review & editing, Methodology, Funding acquisition. **Guilherme A.M. Pasquali:** Writing – review & editing, Methodology, Funding acquisition. **Leon Hall:** Writing – review & editing, Resources, Project administration, Methodology, Funding acquisition. **Tamsyn M. Crowley:** Writing – review & editing, Supervision, Resources, Project administration. **Nishchal K. Sharma:** Writing – original draft, Project administration, Formal analysis.

## Declaration of competing interest

We declare that we have no financial and personal relationships with other people or organizations that can inappropriately influence our work, and there is no professional or other personal interest of any nature or kind in any product, service and/or company that could be construed as influencing the content of this paper.
